# Stress alters the expression of cancer-related genes in the prostate

**DOI:** 10.1186/s12885-017-3635-4

**Published:** 2017-09-05

**Authors:** Ivan E. Flores, Jorge A. Sierra-Fonseca, Olinamyr Davalos, Luis A. Saenz, Maria M. Castellanos, Jaidee K. Zavala, Kristin L. Gosselink

**Affiliations:** Department of Biological Sciences and Border Biomedical Research Center, The University of Texas at El Paso, 500 West University Avenue, El Paso, TX 79968 USA

**Keywords:** Stress, Prostate cancer, HPG axis, Prostate gene expression

## Abstract

**Background:**

Prostate cancer is a major contributor to mortality worldwide, and significant efforts are being undertaken to decipher specific cellular and molecular pathways underlying the disease. Chronic stress is known to suppress reproductive function and promote tumor progression in several cancer models, but our understanding of the mechanisms through which stress contributes to cancer development and progression is incomplete. We therefore examined the relationship between stress, modulation of the gonadotropin-releasing hormone (GnRH) system, and changes in the expression of cancer-related genes in the rat prostate.

**Methods:**

Adult male rats were acutely or repeatedly exposed to restraint stress, and compared to unstressed controls and groups that were allowed 14 days of recovery from the stress. Prostate tissue was collected and frozen for gene expression analyses by PCR array before the rats were transcardially perfused; and brain tissues harvested and immunohistochemically stained for Fos to determine neuronal activation.

**Results:**

Acute stress elevated Fos expression in the paraventricular nucleus of the hypothalamus (PVH), an effect that habituated with repeated stress exposure. Data from the PCR arrays showed that repeated stress significantly increases the transcript levels of several genes associated with cellular proliferation, including proto-oncogenes. Data from another array platform showed that both acute and repeated stress can induce significant changes in metastatic gene expression. The functional diversity of genes with altered expression, which includes transcription factors, growth factor receptors, apoptotic genes, and extracellular matrix components, suggests that stress is able to induce aberrant changes in pathways that are deregulated in prostate cancer.

**Conclusions:**

Our findings further support the notion that stress can affect cancer outcomes, perhaps by interfering with neuroendocrine mechanisms involved in the control of reproduction.

**Electronic supplementary material:**

The online version of this article (10.1186/s12885-017-3635-4) contains supplementary material, which is available to authorized users.

## Background

Stress is a highly complex process that disrupts homeostasis and involves environmental and psychosocial factors acting as stimuli (stressors) to induce a series of responses by the peripheral and central nervous systems [1]. One of the physiological systems that becomes activated during stress is the hypothalamic-pituitary-adrenal (HPA) axis. Activation of the HPA axis triggers the release of corticotrophin-releasing hormone (CRH) from the paraventricular nucleus of the hypothalamus (PVH) which, in turn, induces the anterior pituitary to secrete adrenocorticotropic hormone (ACTH), ultimately resulting in the production of cortisol by the adrenal cortex. The entire axis is then regulated via negative feedback provided by cortisol at the hypothalamic and pituitary levels [2]. Chronic stress can adversely alter hormone levels, thus affecting the regulation of the stress response which can ultimately have a negative effect on the overall physiology of the body [3, 4]. Therefore, stress can have deleterious effects on health, contributing to the incidence and progression of diseases such as cancer.

Chronic stress is, in fact, believed to be a significant factor in the development and progression of cancer, and a growing body of evidence suggests that the neuroendocrine stress response machinery is an important mediator during tumorigenesis and metastasis [5–9]. Impaired T cell-mediated immunity, enhanced tumor proliferation, and decreased survival have been shown in response to chronic stress in a mouse lymphoma model [10]. Suppression of T cell function by chronic stress is also known to increase susceptibility to skin cancer [11], and β-adrenergic signaling has been shown to be linked to tumor growth and invasion in pancreatic and ovarian cancers [12, 13]. Stress-related hormones have been shown to be involved in accelerating cell proliferation and tumor growth in breast and prostate cancer [14, 15], while inhibiting cell growth in other tumors such as neuroblastoma [16], thus indicating that cell type and specific hormones are critical factors in mediating the stress-cancer relationship.

In addition to altering the regulation of the HPA axis, acute and chronic stress can also affect the hypothalamic-pituitary-gonadal (HPG) axis, which can in turn disrupt reproductive function since the hormones within this axis are responsible for orchestrating mammalian reproduction. The first step of the HPG axis is the secretion of gonadotropin-releasing hormone (GnRH) by the preoptic area of the hypothalamus, which then stimulates the release of luteinizing hormone (LH) and follicle-stimulating hormone (FSH) from the anterior pituitary in a fashion that is dependent on the frequency and amplitude of GnRH pulses; the gonadotropic hormones LH and FSH then regulate steroidogenesis and gametogenesis in the gonads [17, 18]. Stress can suppress the HPG axis through inhibiting GnRH secretion which, in turn, suppresses pituitary release of LH and FSH [19, 20]. Chronic restraint stress in rats also reduces GnRH pulsatility and lowers plasma LH and FSH [21]. It should be noted, however, that rebound GnRH secretion can occur following earlier suppression of its release [22–24]. GnRH is strongly implicated as having a role cancer, but conflicting data on the effects of GnRH on cellular function have been published. GnRH analogs inhibit growth and proliferation in reproductive cancer cell lines in vitro [21, 25–28], and also decrease growth in melanoma cells [29], suggesting that GnRH may have broad anti-cancer functions. Furthermore, GnRH receptor agonists are currently being evaluated for potential use in the treatment of prostate, breast, endometrial and ovarian cancers as well as glioblastomas and melanomas, due to their ability to halt cellular proliferation [30]. However, GnRH receptor antagonists may alternatively be used to knock down the effects of endogenous GnRH and inhibit the release of steroids that stimulate tumor growth in certain cancers [31], demonstrating that GnRH does not always produce desired effects. While accumulating evidence seems to support a clear link between stress and cancer, a direct, mechanistic relationship through which stress can alter reproductive function and subsequently contribute to cancer has not been established.

Prostate cancer (PC) is the most commonly diagnosed non-skin malignancy and the second most prevalent cause of cancer death in the U.S., accounting for ~10% of newly diagnosed male cancer cases worldwide [32–34]. Taking current screening habits into consideration, it is predicted that 1 in 6 men alive today in the U.S. will be diagnosed with the disease, and roughly 3% of those patients will die from it [35, 36]. GnRH analogs have been previously used as antineoplastic drugs against PC [37–39]. The use of GnRH agonists is the current choice for androgen-deprivation therapy for advanced and metastatic PC. The rationale behind this treatment is to stimulate the release of LH to achieve feedback regulation of testosterone levels. This method initially leads to a testosterone surge that can worsen the disease, but continuous administration of the agonist eventually downregulates the pituitary GnRH-R, regulating LH release and decreasing testosterone production [40]. On the other hand, GnRH antagonists act by directly binding to GnRH-R, thus avoiding the testosterone “flare” caused by GnRH agonists [41, 42]. Even though it is clear that chronic stress leads to the suppression of GnRH, and GnRH constitutes a target for PC treatment, no study to date has directly examined the relationship between stress-induced modulation of the GnRH system and PC-related outcomes in a single in vivo model. Therefore, we aimed to determine if chronic stress modulates hypothalamic GnRH and consequently affects the expression of genes associated with prostate cell proliferation, and if recovery from stress could induce a rebound of GnRH release that subsequently contributes to an upregulation of genes that promote metastasis.

## Methods

### Experimental animals

Adult male Sprague/Dawley Rats were housed individually in standard cages and maintained on a 12:12 h cycle with food and water ad libitum. Rats were allowed to acclimate for one week before being used for experiments. All animals used in this study were cared for in accordance with the Guide for the Care and Use of Laboratory Animals, and all procedures were approved by the UTEP Institutional Animal Care and Use Committee (IACUC protocol A-201006-1).

### Restraint stress

Rats were randomly assigned to 7 groups (*n* = 5/group): Control (Con), Acute restraint (Acu), Repeated restraint (Rep), Control plus recovery (Con + Rec), Acute restraint plus recovery (Acu + Rec), Repeated restraint plus recovery (Rep + Rec). A final group underwent repeated restraint and recovery followed by an additional acute restraint exposure (R + A). Physical restraint was used as an emotional stressor, with the rats placed inside an acrylic restraining device (Kent Scientific) for 30 min. All restraint and exposure was done near the beginning of the light cycle, between 0900 and 1100 h. Acu rats were exposed to open restraining devices in their home cages for 30 min/d for 20 consecutive days, and then restrained for 30 min on the 21st day only, while Rep rats were restrained for 30 min on each of the 21 days. Con rats were exposed to the restraining device daily for 30 min but never restrained. Rats in the recovery groups were handled identically to their Con, Acu or Rep counterparts, but then allowed 14d of recovery with no manipulation after their stress exposure. Rats in the final group (R + A) were restrained for 30 min/d for 21d, free from stress exposure for 13d, and finally restrained for 30 min on the last day.

### Perfusion and tissue collection

At the end of the restraining treatments, the animals were deeply anesthetized with 100 mg/kg of sodium pentobarbital, i.p. (Nembutal®; McKesson), followed by perfusion through the ascending aorta with ~100 mL of 0.9% saline, and 400–500 mL of 4% paraformaldehyde (JT Baker) at pH 9.5 in 0.1 M borate buffer. Brains were dissected, post-fixed for 5 h at 4 °C, and cryoprotected overnight at 4 °C in KPBS with 10% sucrose, then serially sectioned in 30 μm sections on a freezing microtome (Model SM 2000R; Leica) and stored in antifreeze (30% ethylene glycol, 20% glycerol) at −20 °C until used for immunohistochemical analysis. Prostate tissues were dissected during the saline and tissue samples (ranging from 15 to 30 mg) were obtained, placed on dry ice, and stored at −80 °C until use for transcriptional analysis.

### Immunohistochemistry and cell counting

Brain sections were immunohistochemically stained for Fos and GnRH as an indicator of neuronal activation in response to stress. Fos was localized using a nickel-intensified avidin-biotin-immunoperoxidase technique. Tissue sections were incubated overnight at 4 °C in primary antiserum against Fos (1:10,000; Oncogene Science), and incubated on the following day for 1 h at room temperature in secondary antibody (biotinylated goat anti-rabbit IgG, 1:200; Vector). An avidin-biotin-complexing solution (Vectastain Elite kit; Vector) was applied for 1 h, and a nickel-enhanced glucose oxidase method using diaminobenzidine (DAB) as a chromogen was used to visualize specific binding. Separate series of brain sections were immunostained for GnRH using a similar method but without the nickel enhancement (primary antiserum used at 1:10,000; Abcam). Stained sections were mounted on gelatin-coated slides, dehydrated through a graded series of ethanol and xylene, and coverslipped with DPX mountant (Electron Microscopy Sciences). The number of Fos-positive cells in the PVH was quantified by simple cell counting on a light microscope coupled to a digital imaging system (Zeiss). Counts were taken unilaterally from 4 sections throughout the rostrocaudal extent of the PVH, and summed for each animal. Group averages were then calculated, and compared statistically using two-tailed t-tests with a *p* value of ≤0.05 considered significant.

### Transcriptional analysis

Total RNA was isolated from prostate tissue samples (15–30 mg) using a commercially available kit (RNeasy mini kit, Qiagen) according to manufacturer’s instructions. RNA concentration and purity were determined using a Nanodrop spectrophotometer and samples were stored at −20 °C until use. cDNA was generated using a RT^2^ first strand kit and used in two different targeted PCR arrays (SABiosciences): one array for genes associated with cancer pathways (Cancer Pathway Finder PARN-033A) and another one for genes associated with tumor metastasis (Tumor Metastasis PARN-028A). For the cancer pathway array, an n of 5 was evaluated for all groups except Con + Rec (*n* = 4) and R + A (*n* = 2); in the metastasis array an n of 3 per group was used except for Con + Rec (*n* = 2). Gene expression changes were analyzed by means of the ΔCt (cycle threshold) method [43], subtracting the average Ct values obtained from the housekeeping genes provided in the array from the average Ct values of every gene of interest present in the arrays. Only Ct values below 35 were used in the study, as determined by the quality control reactions included in the array. Statistical analysis on the ΔCt experimental values was performed using Microsoft Excel and Sigma Plot 12.5 software, with a statistical significance of *p* ≤ 0.05 determined by two-tailed t-test for between group comparisons. Further testing employed one-way ANOVA with Bonferroni correction to account for multiple comparisons and to determine overall effects.

### Molecular function and biological process of genes with altered expression

In order to identify the cellular and molecular functions of the genes with significant changes in expression, the UniProtKB Knowledgebase [44] was used. Gene IDs from the arrays (as provided by the manufacturer, SABiosciences) were entered into the UniProtKB search engine, and only search results from rat were consulted. The information from GO-molecular function and GO-biological process was used to identify the function of the genes and the biological processes in which they participate. Preference was given to those results that were derived from experimental evidence, as indicated by the database.

## Results

### Effects of restraint stress on hypothalamic neuroendocrine pathways

**Fig. 1:**
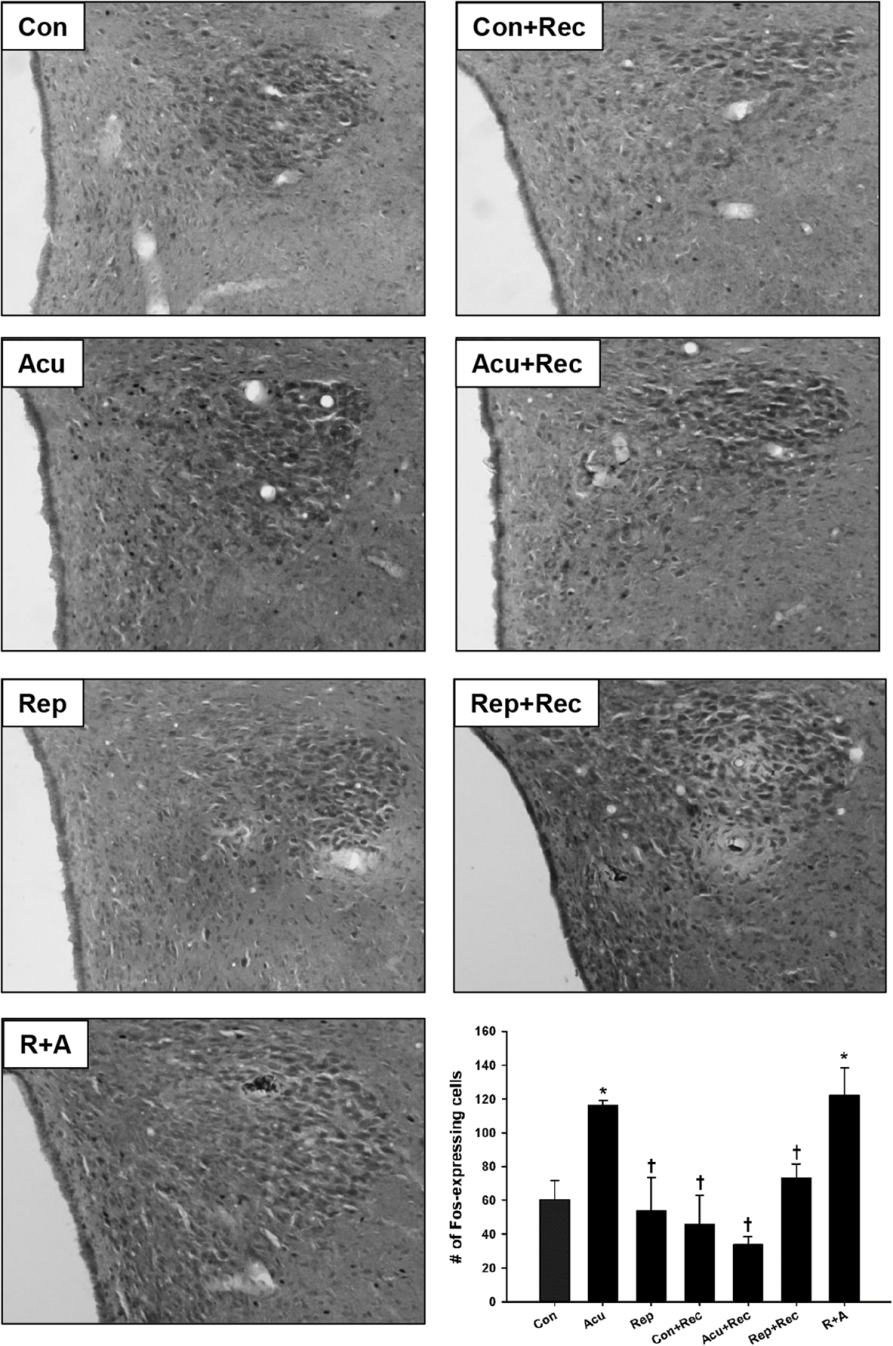
Stress-induced expression of Fos in the PVH. Representative photomicrographs are provided for PVH sections from each treatment group immunohistochemically stained for Fos. The bar graph displays quantitative results of the total number of Fos-positive cells in Control (Con), Acutely stressed (Acu), Repeatedly Stressed (Rep), Control plus Recovery (Con + Rec) Acute plus Recovery (Acu + Rec), Repeated plus Recovery (Rep + Rec), and Repeated plus Recovery plus Acute (R + A) animals. Bars are Mean ± SEM. *n* = 3 for each group, except Rep where *n* = 4. Symbols: *, significantly different (*p* < 0.05) from Con; †, significantly different (*p* < 0.05) from Acu

Brain tissue sections containing the PVH were immunohistochemically stained for Fos as a marker of neuronal activation following restraint stress and recovery. The number of Fos-expressing cells increased significantly following acute stress when compared to control (Fig. 1). This effect habituated with repeated stress exposure, as counts of Fos-positive neurons in the PVH in repeatedly stressed rats (Rep) were not different from those seen in Con rats. The 14-day recovery period post-stress ameliorated the Fos response in Acu rats, such that the number of Fos-positive neurons returned control levels.

Separate sections of tissue were stained for Fos and GnRH, and singly- and doubly-labeled cells quantified in the medial preoptic area. We were able to identify small numbers of neurons that expressed GnRH and were activated by stress (Fos-positive), but no statistically significant differences were found by treatment group (data not shown).

### Acute and repeated restraint stress upregulate cancer pathway-associated genes in the rat prostate

Transcriptional analysis of prostate tissue from rats subjected to Acu restraint stress revealed a group of seven genes in the cancer pathway platform that displayed significantly upregulated expression (Table 1). This same set of genes remained significantly elevated in their expression following Rep stress, with the exception of one gene (Arnt, Aryl hydrocarbon receptor nuclear translocator, a transcription factor) which returned to control-like levels in the Rep group. A recovery period (no restraint) of 14 days post-Rep stress was sufficient to return the expression of these genes to normal (control) levels. Within this group of genes, Ets2 (V-ets erythroblastosis virus E26 oncogene homolog 2) and Skp2 (S-phase kinase-associated protein 2, p45) were identified as proto-oncogenes, and Krt14 (Keratin 14) is a structural protein that is widely used as a marker for prostate adenocarcinoma.

**Table 1:** Cancer pathway genes with significant upregulation after Acu and Rep stress, returning to normal after recovery

Gene ID/ Accesion	Gene name	Fold change Con vs Acu	Fold change Con vs Rep	Molecular function/biological process
*Arnt P41739*	*Aryl hydrocarbon receptor nuclear translocator*	*1.51*	*1.23 (ns)*	*Transcription factor/response to hypoxia*
**Ets2 D4AAH4**	**V-ets erythroblastosis virus E26 oncogene homolog 2 (avian)**	**1.83**	**1.93**	**Transcription factor/cell differentiation**
Krt14 Q6IFV1	Keratin 14	2.27	2.27	Cytoskeleton/maintenance of cell morphology
Serpinb2 P29524	Serpin peptidase inhibitor, clade B (ovalbumin), member 2	1.97	1.99	Endopeptidase inhibitor/apoptosis
**Skp2 B2GUZ0**	**S-phase kinase-associated protein 2 (p45)**	**1.56**	**1.67**	**Ubiquitin ligase/cell cycle regulation**
Slc2a1 P11167	Solute carrier family 2 (facilitated glucose transporter, member 1	1.25	1.32	Glucose transporter/metabolism (nutrient uptake)
Tinf2 Q5XIB8	TERF 1 (TRF1)- interacting nuclear factor 2	4.29	6.11	Telomeric DNA binding/chrosomome stability

Italicized text denotes a gene that was only upregulated with Acu (but not Rep) stress, as indicated by “ns”; bold text denotes genes identified as proto-oncogenes. Accession numbers and molecular function/biological process obtained from UniProtKB database

A different set of 25 genes were upregulated after Rep stress, but did not show significant changes when subjected to Acu stress (Table 2), suggesting that transient stress exposure is not sufficient to change their expression levels. These genes varied in function from cell cycle regulation, to metabolism, to DNA repair and cell adhesion. Importantly, 2 of these genes, Fms-related tyrosine kinase 1 (Flt1) and Mitogen activated protein kinase kinase 1 (Map2k1), were both identified as proto-oncogenes involved in mitogenic cell signaling. Also worth noting was the finding that about one third of these genes participate in apoptosis. Expression levels and statistical evaluation of genes in the Cancer Pathway platform are included as Additional files (Additional files 1 and 2).

**Table 2:** Cancer pathway genes significantly upregulated after Rep stress (but not after Acu)

Gene ID/ Accession	Gene name	Fold change Con vs Rep	Molecular function/biological process
Acly P16638	ATP citrate lyase	2.14	Lyase enzyme/metabolism (bioenergetics)
Angpt1 O35460	Angiopoietin 1	1.91	Vascular growth factor/angiogenesis
Apaf1 Q9EPV5	Apoptotic peptidase activating factor 1	1.42	Endopeptidase/apoptosis
Atp5a1 P15999	ATP synthase, H+ transpoting, mitochondrial F1 complex, alpha subunit	1.84	ATP synthesis/metabolism (bioenergetics)
Casp2 P55215	Caspase 2	2.10	Endopeptidase/apoptosis
Casp7 O88550	Caspase 7	1.87	Endopeptidase/apoptosis
Casp9 Q920G4	Caspase 9, apoptosis-related cysteine peptidase	1.96	Endopeptidase/apoptosis
Ccnd3 P48961	Cyclin D3	1.54	Kinase/cell cycle regulation
Cdc20 Q62623	Cell division cycle 20 homolog (*S. cerevisiae*)	1.92	Regulatory protein/cell cycle regulation
Cdh2 Q9Z1Y3	Cadherin 2	3.07	Cell adhesion
Cflar C0H5Y5	CASP8 and FADD-like apoptosis regulator	2.08	Endopeptidase/apoptosis
Cpt2 P18886	Carnitine palmitoyltransferase 2	2.06	Lipid transferase/metabolism (fatty acid)
Dkc1 P40615	Dyskeratosis congenita 1, dyskerin	1.66	rRNA processing/proliferation
E2f4 D4A9V4	E2F transcription factor 4	2.10	Transcription factor/proliferation
Ercc5 D3ZTV2	Excision repair cross-complementing rodent repair deficiency, Complementation group 5	1.85	Endonuclease/DNA excision repair
**Flt1 P53767**	**Fms-related tyrosine kinase 1**	**2.77**	**Growth factor receptor/proliferation, migration**
Foxc2 Q63246	Forkhead box C2	1.91	Transcription factor/proliferation, differentiation
G6pd P05370	Glucose −6-phosphate dehydrogenase	2.38	Dehydrogenease/metabolism (carbohydrates)
Igfbp7 Q5RJM3	Insulin-like growth factor binding protein 7	1.89	Growth factor binding/response to hormones
**Map2k1 Q01986**	**Mitogen activated protein kinase kinase 1**	**1.83**	**Mitogenic protein kinase/proliferation**
Nol3 Q62881	Nucleolar protein 3 (apoptosis repressor with CARD domain)	2.11	Regulatory protein/apoptosis
Pgf Q63434	Placental growth factor	1.60	Growth factor/proliferation, differentiation
Pp1r15a Q6IN02 Ppp1r15a	Protein phosphatase 1, regulatory (inhibitor) subunit 15A	1.91	Phosphatase/apoptosis
Sod1 P07632	Superoxide dismutase 1, soluble	1.96	Dismutase enzyme/oxidative stress
Wee1 Q63802	Wee 1 homolog (S. pombe)	1.29	Kinase/cell cycle regulation

Bold text denotes proto-oncogenes. Accession numbers and molecular function/biological process obtained from UniProtKB database

### Restraint stress differentially affects the expression of prostate genes affiliated with the metastatic program

Acu stress significantly upregulated the expression of only three genes known to be involved in tumor metastasis (Table 3), which were fibroblast growth factor receptor 4, heparanase, and integrin beta 3. The expression of these genes was not increased under Rep stress conditions, however, and their expression levels returned to control values when allowed to recover from Acu stress, suggesting that the observed upregulation induced by Acu stress could be a transient effect, as seen previously with Arnt (Table 1).

**Table 3:** Metastasis-associated genes with significant upregulation after Acu stress (but not Rep), returning to normal after recovery

Gene ID/ Accession	Gene name	Fold change Con vs Acu	Molecular function/ biological process
Fgfr4 Q498D6	Fibroblast growth factor receptor 4	3.32	Growth factor receptor/ proliferation
Hpse Q71RP1	Heparanase	2.75	Endoglycosidase/ cell adhesion
Itgb3 Q8R2H2	Integrin, beta 3	2.45	Receptor protein/ cell adhesion, angiogenesis

Accession numbers and molecular function/biological process obtained from UniProtKB database

Rep stress was able to induce the significant upregulation of several genes in the metastasis array (Table 4), one of which (Src) is a very well-established proto-oncogene that participates in promoting cell proliferation. Although the expression levels of the majority of these genes decreased when the rats were allowed to recover from Rep stress, the expression of two genes (Chemokine (C-C motif) ligand 7 and Plasminogen activator, urokinase receptor) did not fully return to control levels, indicating a persistent effect of Rep stress. In addition to increasing the expression of the genes mentioned above, Rep stress significantly downregulated the expression of p53, a very well characterized tumor suppressor gene. Importantly, the expression of p53 remained significantly downregulated (although not to the extent observed after Rep stress) even when allowed to recover from stress. Complete datasets containing expression levels and statistical evaluation of genes in the Metastasis platform are included as Additional files (Additional files 3 and 4).

**Table 4:** Metastasis-associated genes that displayed significant changes in expression after Rep stress (but not Acu)

Gene ID/ Accession	Gene name	Fold change Con vs Rep	Molecular function/biological process
*Ccl7 Q9QXY8*	*Chemokine (C-C motif) ligand 7*	*11.00*	*Cytokine/immune function (chemotaxis)*
Il1b Q63264	Interleukin 1 beta	3.84	Cytokine/immune function (inflammation)
*Plaur P49616*	*Plasminogen activator, urokinase receptor*	*6.63*	*Transmembrane protein/differentiation*
**Src Q9WUD9**	**V-src sarcoma**	**1.93**	**Non-receptor tyrosine kinase/proliferation**
Tp53* P10361	***Tumor protein p53***	***0.50***	***Transcription factor/DNA repair, apoptosis***

Italicized text denotes genes that remained significantly changed after recovery. Only one gene was found to have downregulated expression (asterisk); bold text denotes genes identified as proto-oncogenes; bold and italicized text denotes tumor suppressor genes. Accession numbers and molecular function/biological process obtained from UniProtKB database

## Discussion

Accumulating evidence provided by epidemiological studies strongly suggests that chronic psychological stress plays an important role in the initiation and progression of cancer [45–47]. Results from clinical studies also strongly support a link between stressful events in a patient’s life with reduced cancer survival [46, 48]. However, the precise mechanisms by which chronic stress influences tumorigenesis and carcinogenesis remain poorly understood. Although much progress has been made through the use of animal models, most of these studies have used mouse xenograft and genetic models to study the effects of chronic stress (such as restraint and social isolation) on cancer progression and metastasis [12, 49–52].To the best of our knowledge, this is the first study designed to identify changes in the expression of cancer-associated genes as a response to chronic emotional stress in a specific organ of an otherwise healthy animal. This allowed us to determine changes in prostate gene expression in response to chronic stress compared to basal levels, which has provided us with insights as to which cellular pathways can be initially disrupted by stress that may lead to abnormal cell proliferation and tumor formation.

Our results demonstrate that restraint stress (both acute and chronic) is sufficient to alter the expression of genes associated with tumor proliferation and metastasis in the rat prostate. Previous studies using a rat model of chronic stress (by restraint water immersion) have shown that 14 days of stress can induce histological changes in the prostate, as well as proliferation of epithelial cells of the ventral lobe of the prostate [53, 54]. Although the expression of proliferation-associated genes was not measured in these studies, our data supports the notion that restraint stress can induce aberrant gene expression, which could potentially lead to increased cell proliferation. The genes included in this investigation have broad cellular functions and their up and downregulation may not necessarily indicate cancer development or progression in this model. However the sensitivity of these genes to stress provides evidence of potential mechanisms through which increased stress exposure might ultimately contribute to cancer.

Although PC is usually considered a disease of aged men (~70% of diagnosed patients are over 65), and age itself is one of the major risk factors in developing the disease [36], histopathological studies using prostate tissue samples from younger healthy patients (age 20–40) have demonstrated the presence of proliferative lesions, indicating that cancer initiation may take place at an early age and remain undetected for many years [55–58]. Our data demonstrate aberrant expression of cancer-related genes in the tissues of adult animals in response to chronic stress. It is therefore reasonable to speculate that chronic stress may either trigger localized cell proliferation at an early age, which can then accumulate additional “hits” over time until progressing into full fledged cancer. Another interesting possibility is that chronic stress may serve to aggravate pre-existing neoplastic lesions, thus contributing to cancer progression.

Despite the lack of knowledge regarding specific mechanisms underlying tumorigenic processes induced by stress, previous studies have identified several physiological and molecular pathways that are influenced by stress and can contribute to cancer progression. Several studies have focused on immune function, since it is very well established that stress can downregulate immune function, which in turn can impair the immune response against tumor cells [59–61]. Importantly, it was recently shown that chronic stress can induce a re-organization of lymphatic vessels to facilitate the dissemination of tumor cells in a mouse model of breast cancer, and this was proven to be regulated by the sympathetic nervous system [62]. However, decreased immune function alone cannot account for the multitude of processes that need to be altered in order for cancer to progress. One major contributor is the neuroendocrine response to stress, including the HPA and HPG axes, which trigger hormone signaling descending from the brain and the pituitary to the adrenal glands and reproductive tissues. These systems can have myriad effects on peripheral tissues, including neurotransmitter release and production of mitogenic factors that can promote tumor growth by acting directly on receptors present in cancer cells [6], as well as suppression of hormone pulses that can potentially promote cell proliferation, such as GnRH.

In the case of our study, the cell counts of activated neurons in the PVH, as identified by Fos immunostaining, provide evidence that acute restraint stress is activating the HPA axis. This response is well-established, and involves an increase in the neuroendocrine secretion of corticotropin-releasing factor and, ultimately, enhanced release of the glucocorticoid hormone cortisol. Of our genes displaying upregulated expression levels in the Acu condition, a number of them are regulated by cortisol or have related functions. Krt14, for example, is one of many glucocorticoid-regulated keratin genes that tend to be repressed in skin following cortisol-receptor monomers binding to glucocorticoid response elements in the presence of corticosteroid binding protein [63]. Our observed increase in Krt14 expression may be tissue-specific or a function of alternate binding strategies. Increases in Arnt and Fgfr4 may directly result from increased cortisol exposure. The aryl hydrocarbon receptor and glucocorticoid receptor have been shown previously to interact and participate in complex developmental and physiological processes [64]. Cortisol has been shown to increase Fgfr4 expression, and this molecule has been specifically associated with extracellular matrix degradation and tumor invasion in prostate and other cancers [65]. Metabolically, the glucoregulatory capacity of cortisol provides another link to Fgfr4 which also functions in glucose regulation [65], and to Slc2a1, a facilitated glucose transporter that showed upregulated expression after acute stress in our study. Lastly, prostate cancer patients have been shown to have increased circulating levels of cortisol [66], suggesting a possible bi-directional relationship between this stress hormone and cancer. Future experiments will focus on identifying the effects of our stress paradigm on different players within the HPG axis.

The broad molecular functions and biological processes affected by the genes that displayed significantly altered expression levels in our study underlie the observation that stress can affect a multitude of cellular functions that can ultimately lead to increased cell proliferation, including apoptosis, mitogenic signaling, extracellular matrix, DNA repair, and altered bioenergetics. This is not surprising, as cancer is a highly complex, multistep process that requires cells to acquire specific traits in order to proliferate and evolve into a malignant phenotype, and though it is unlikely that stress alone can provide cells with such specific characteristics, it is certainly possible that, given the progressive nature of cancer, stress can facilitate deregulation of certain pro- and anti-proliferative cellular processes [6, 67]. Interestingly, our results showed that restraint stress upregulated the expression of genes involved in the activation of apoptotic pathways (i.e. caspases 2, 7, and 9) and are thus associated with anti-proliferative activity. Recent studies have suggested that psychological stress can promote prostate carcinogenesis in mouse xenograft models via inhibition of apoptosis [68, 69]. While this finding appears to conflict with our gene expression data, it is also possible that our observed increase in the expression of pro-apoptotic genes could be a compensatory response by the prostate cells since we also observed an upregulation of genes that promote cell proliferation (i.e. cyclin D3, Src, MAPK). In fact, prostate tissue is known to be very sensitive to signals that influence tumorigenic growth, and therefore maintains a cell death/proliferation equilibrium [70, 71].

Of noteworthy importance is the upregulated expression of several genes identified as proto-oncogenes, including genes with very well characterized roles in cancer progression such as Map2k1 and Src. Importantly, the expression of tumor protein p53, one of the most characterized tumor suppressor genes, was found to be downregulated in accordance with its cellular role in cancer where inactivation of this gene is sufficient to predispose individuals to cancer [72, 73]. Moreover, chronic stress has been found to promote tumorigenesis in a p53 genetic mouse model of cancer, with tumor formation depending on glucocorticoid action [52].

## Conclusions

The results of the present study illustrate the effects of emotional stress on prostate tissue gene expression. As shown by the functions and processes in which the affected genes participate, stress can influence a wide variety of cellular functions, and may be an early stimulus that promotes prostate cancer incidence or progression through modification by endocrine mediators. That recovery from stress is able to restore the expression of some genes, but not others, to normal levels provides support for future investigations into the importance of stress timing and duration in cancer diagnoses.

## Additional files

Additional file 1: Expression levels of genes in the Cancer Pathway Finder array. Values displayed include mean ΔCT, along with SEM, confidence intervals, and *p* values derived from t-tests for the corresponding comparisons. Samples with insufficient material are indicated as not determined (N/D), and *p* values are not provided for these comparisons. (XLSX 28 kb)

Additional file 2: Statistical evaluation of cancer pathway gene expression by ANOVA with Bonferroni post-hoc analysis to determine overall effects of our treatment paradigm. For genes in which a significant main effect (F-test) was seen, adjusted *p* values are provided. Samples with insufficient material are indicated as not determined (N/D), and *p* values are not provided for these comparisons. (XLSX 15 kb)

Additional file 3: Expression levels of metastasis-associated genes in the Tumor Metastasis array. Values displayed include mean ΔCT, along with SEM, confidence intervals, and *p* values derived from t-tests for the corresponding comparisons. Samples with insufficient material are indicated as not determined (N/D), and *p* values are not provided for these comparisons. (XLSX 29 kb)

Additional file 4: Statistical evaluation of metastasis gene expression by ANOVA with Bonferroni post-hoc analysis to determine overall effects of our treatment paradigm. For genes in which a significant main effect (F-test) was seen, adjusted *p* values are provided. Samples with insufficient material are indicated as not determined (N/D), and *p* values are not provided for these comparisons. (XLSX 14 kb)

## Abbreviations

ACTH: adrenocorticotropic hormone
CRH: corticotrophin-releasing hormone
CT: cycle threshold
DAB: diaminobenzidine
FSH: follicle-stimulating hormone
GnRH: gonadotropin-releasing hormone
GnRH-R: gonadotropin-releasing hormone receptor
HPA: hypothalamic-pituitary-adrenal
HPG: hypothalamic-pituitary-gonadal
LH: luteinizing hormone
PC: prostate cancer
PVH: paraventricular nucleus of the hypothalamus
